# **Retraction notice for:** “miR-141 is negatively correlated with TLR4 in neonatal sepsis and regulates LPS-induced inflammatory responses in monocytes” [Braz J Med Biol Res (2021) 54(7): e10603]

**DOI:** 10.1590/1414-431X2023e10603retraction

**Published:** 2023-09-22

Xinyu Lin^1^ https://orcid.org/0000-0002-5780-0645 and Yaohui Wang^2^ https://orcid.org/0000-0002-3401-334X

^1^Neonatology Department, Weifang People’s Hospital, Weifang, China

^2^Occupational Disease Department, Weifang People’s Hospital, Weifang, China

Correspondence: Yaohui Wang: <sunnyma0101@163.com>

Retraction notice for: Braz J Med Biol Res | doi: 10.1590/1414-431x2020e10603 | PMID: 34008755 | PMCID: PMC8130060

The authors requested retraction of the article “miR-141 is negatively correlated with TLR4 in neonatal sepsis and regulates LPS-induced inflammatory responses in monocytes” that was published in volume 54 no. 7 (2021) (Epub May 17, 2021) in the Brazilian Journal of Medical and Biological Research.

The corresponding author stated the following: “In recent studies, we found that the previous cell experiments in the published article could not be replicated. As a result, we have doubts about the accuracy of our published results".

The Editors decided to retract this paper to avoid further damage to the scientific community. The Brazilian Journal of Medical and Biological Research remains vigilant to prevent misconduct and reinforces the Journal’s commitment to good scientific practices.

